# Effect of resveratrol on insulin action in primary myotubes from lean individuals and individuals with severe obesity

**DOI:** 10.1152/ajpendo.00299.2023

**Published:** 2024-02-07

**Authors:** Sanghee Park, Filip Jevtovic, Polina M. Krassovskaia, Alec B. Chaves, Donghai Zheng, Jonas T. Treebak, Joseph A. Houmard

**Affiliations:** ^1^Human Performance Laboratory, East Carolina University, Greenville, North Carolina, United States; ^2^Department of Kinesiology, East Carolina University, Greenville, North Carolina, United States; ^3^East Carolina Diabetes and Obesity Institute, East Carolina University, Greenville, North Carolina, United States; ^4^Department of Exercise Rehabilitation, Gachon University, Incheon, Republic of Korea; ^5^Section of Integrative Physiology, Novo Nordisk Foundation Center for Basic Metabolic Research, Faculty of Health and Medical Sciences, University of Copenhagen, Copenhagen, Denmark

**Keywords:** glucose metabolism, human skeletal muscle, insulin signaling, resveratrol, obesity

## Abstract

Resveratrol, a natural polyphenol compound contained in numerous plants, has been proposed as a treatment for obesity-related disease processes such as insulin resistance. However, in humans there are conflicting results concerning the efficacy of resveratrol in improving insulin action; the purpose of the present study was to determine whether obesity status (lean, severely obese) affects the response to resveratrol in human skeletal muscle. Primary skeletal muscle cells were derived from biopsies obtained from age-matched lean and insulin-resistant women with severe obesity and incubated with resveratrol (1 µM) for 24 h. Insulin-stimulated glucose oxidation and incorporation into glycogen, insulin signal transduction, and energy-sensitive protein targets [AMP-activated protein kinase (AMPK), Sirt1, and PGC1α] were analyzed. Insulin-stimulated glycogen synthesis, glucose oxidation, and AMPK phosphorylation increased with resveratrol incubation compared with the nonresveratrol conditions (main treatment effect for resveratrol). Resveratrol further increased IRS1, Akt, and TBC1D4 insulin-stimulated phosphorylation and SIRT1 content in myotubes from lean women, but not in women with severe obesity. Resveratrol improves insulin action in primary human skeletal myotubes derived from lean women and women with severe obesity. In women with obesity, these improvements may be associated with enhanced AMPK phosphorylation with resveratrol treatment.

**NEW & NOTEWORTHY** A physiologically relevant dose of resveratrol increases insulin-stimulated glucose oxidation and glycogen synthesis in myotubes from individuals with severe obesity. Furthermore, resveratrol improved insulin signal transduction in myotubes from lean individuals but not from individuals with obesity. Activation of AMPK plays a role in resveratrol-induced improvements in glucose metabolism in individuals with severe obesity.

## Graphical abstract

**Figure:**
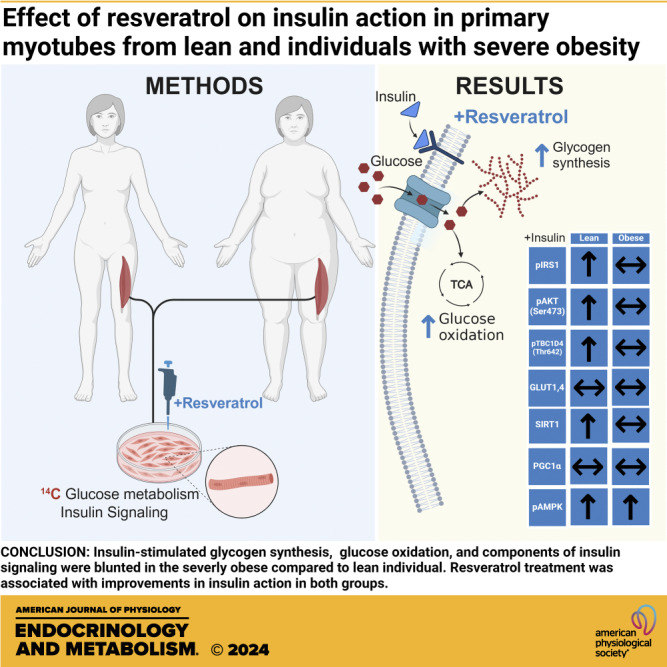

## INTRODUCTION

Obesity is linked with metabolic diseases such as insulin resistance, type 2 diabetes (T2D), and cardiovascular disease. Moreover, the prevalence of severe obesity (BMI ≥ 40 kg/m^2^) has exponentially increased since 2000 in the United States (1, 2), leading to a parallel increase in obesity-related diseases (3). Based on current trends, it is projected that 4 million individuals will be diagnosed with T2D by 2030 (4). Therefore, designing therapeutic targets/interventions for the treatment of obesity and obesity-related disease should be a priority.

Resveratrol may be a candidate for the treatment of obesity-related conditions (5, 6) as it elicits both exercise mimicking (7) and caloric restriction-like effects (8), and it has been demonstrated to improve glucose metabolism in rodents (5, 9–11) and humans (7) with obesity. A prominent feature of resveratrol is its ability to activate energy and redox-sensitive proteins such as AMP-activated protein kinase (AMPK), SIRT1, and PGC1α (5, 9), all of which have been shown to play an important role in insulin-mediated glucose metabolism (12). In animal studies, small doses of resveratrol improved glucose uptake in the absence or presence of insulin (8, 13). Similarly, Timmers et al. (7) reported that 30 days of low-dose (∼1.5 mg/kg/d) resveratrol treatment in humans with obesity significantly reduced fasting plasma glucose and insulin levels. In addition, the resveratrol intervention significantly increased the expression of AMPK, SIRT1, PGC1α, and mitochondrial respiration in skeletal muscle. Although these findings provide meaningful insights into the effects of resveratrol in humans, it is still unclear what role it plays in regulating peripheral insulin action, which is predominantly controlled at the level of the skeletal muscle.

The purpose of this investigation was to determine the effects of resveratrol treatment on insulin action in skeletal muscle cells derived from lean individuals and individuals with severe obesity. Primary human myotubes maintain the metabolic phenotype of the donor; for example, we have reported that myotubes derived from individuals with severe obesity are insulin resistant and exhibit decrements in fatty acid oxidation (14–16). Similarly, others have shown that insulin resistance of the donor is retained in myotubes raised in culture (17–19). This model avoids interference from other tissues and cell types, which allows a specific examination of skeletal muscle cells. We specifically examined insulin action and factors that influence insulin sensitivity and can be altered with resveratrol treatment such as the distal components of the insulin signaling cascade, SIRT1 and PGC1α abundance, and AMPK phosphorylation.

## RESEARCH DESIGN AND METHODS

### Study Design

Human skeletal muscle myotubes derived from lean individuals and individuals with severe obesity were compared in response to 24 h of resveratrol treatment. A physiological concentration of resveratrol was selected based on average blood resveratrol concentrations after intervention in humans (7). The primary functional outcomes were indices of insulin action (insulin-stimulated glycogen synthesis and glucose oxidation). Components contributing to insulin-stimulated metabolism (distal steps of insulin signal transduction, GLUT content) and indices of resveratrol-responsive metabolism were examined as possible means to explain differences between the groups. Lean individuals (BMI < 25 kg/m^2^) and individuals with severe obesity (BMI ≥ 40 kg/m^2^) were recruited. All participants were sedentary (no structured exercise for the previous 6 mo) and provided written informed consent prior to any experimental procedures. Physical activity levels were not obtained. All procedures were approved by East Carolina University Policy and Review Committee on Human Research. Data from some of these myotubes have been presented elsewhere (20).

### Primary Skeletal Muscle Cell Culture

As described previously (21), satellite cells were isolated from muscle biopsies obtained from the vastus lateralis, proliferated to myoblasts, and amplified on type-I collagen-coated plates until reaching ∼80–90% confluence in growth media (DMEM low-glucose medium supplemented with 10% FBS, 0.5 mg/mL BSA, 0.05% fetuin, 20 ng/mL human epidermal growth factor, 0.39 µg/mL dexamethasone, and 100 µg/mL penicillin/streptomycin) in a 5% CO_2_ and 37°C humidified atmosphere. Upon reaching 80–90% confluence, myoblasts were differentiated to myotubes by switching to differentiation media (DMEM low-glucose medium supplemented with 2% horse serum, 0.3% BSA, 0.05% fetuin, and 100 µg/mL penicillin/streptomycin). Experiments were conducted on *days 5* to *6* of differentiation of skeletal muscle cells in *passage 3* or *4*.

### Resveratrol Treatment

Resveratrol (Thermo Fisher Scientific, Waltham, MA) was dissolved in dimethyl sulfoxide (DMSO) (Thermo Fisher Scientific, Waltham, MA). Mature myotubes were incubated in 0.025% DMSO without (control) or with resveratrol (treatment) for 24 h in serum-starvation media (DMEM low-glucose medium supplemented with 1% BSA and 100 µg/mL penicillin/streptomycin). Initial dose-response experiments were conducted in myotubes from lean subjects and indicated that a 24-h, 1 µM resveratrol incubation increased insulin-stimulated glycogen synthesis and glucose oxidation (data not shown). Moreover, this concentration was physiologically appropriate to investigate the effect of resveratrol in humans (7).

### Glycogen Synthesis

As previously described (22), following 24 h of resveratrol incubation, myotubes were incubated with media containing D-[U-^14^C] glucose (Perkin-Elmer, Waltham, MA) (1 µCi/mL, 5.0 mM glucose) in the presence or absence of 100 nM insulin for 2 h at 37°C. Myotubes were then washed twice with ice-cold DPBS and solubilized with 0.05% SDS. Lysates were combined with carrier glycogen (2 mg) and hydrolyzed at 100°C for 1 h, followed by cooling on ice for 30 min. The remaining lysate was used to measure protein concentration using the bicinchoninic acid (BCA) assay (Pierce Biotechnology, Rockford, IL). Ice-cold 100% ethanol was added to the hydrolyzed lysates and precipitated, and samples were rotated overnight at 4°C. Glycogen pellets were then centrifuged at 11,100 *g* for 15 min at 4°C and washed with 70% ethanol followed by centrifugation. The glycogen pellets were resuspended with dH_2_O and incorporation rate from radioactive glucose into glycogen was determined via liquid scintillation.

### Glucose Oxidation

Following the 24-h resveratrol incubation, myotubes were incubated in a sealed plate with media containing D-[U-^14^C] glucose (1 µCi/mL, 5.0 mM glucose) in the presence or absence of 100 nM insulin for 2 h at 37°C. Immediately after incubation, radioactive media were transferred into a customized 48-well trapping plate with fabricated grooves between two adjoining wells to allow for acid-driven ^14^CO_2_ to be trapped by fresh 1 M sodium hydroxide (NaOH) (23). Incorporation of radioactive glucose into CO_2_ was determined with liquid scintillation. Myotubes were washed with ice-cold PBS and solubilized in 0.05% SDS to measure protein concentration.

### Western Blot Analyses

Following 24 h of resveratrol incubation, myotubes were incubated in the presence or absence of 100 nM insulin for 10 min. Myotubes were washed with ice-cold DPBS, harvested in ice-cold lysis buffer containing 50 mM HEPES (JT Becker, Inc., Carnegie, PA), 12 mM sodium phosphate, 100 mM sodium fluoride, 100 mM EDTA, 10 mM sodium orthovanadate, 1% Triton X-100, and protease and phosphatase (1 and 2) inhibitor cocktails (Sigma-Aldrich, St. Louis, MO) followed by a sonication for 5 s. Supernatants were used for immunoblot analysis as previously described (24). Primary antibodies were IRS1(Tyr632) (1:500, 09–433; Millipore, Billerica, MA); IRS1 protein (1:500, sc-559; Santa Cruz Biotechnology, Dallas, TX); Akt (Ser473) (1:1,000, 9271; Cell Signaling); Akt protein (1:1,000, 9272; Cell Signaling); AMP-activated protein kinase (AMPK) (Thr172) (1:1,000, 2531; Cell Signaling); AMPK protein (1:1,000, 2532; Cell Signaling); Akt-substrate at 160 kDa (AS160/TBC1D4) (Thr642) (1:1,000, ab59173; Abcam, Cambridge, MA); AS160/TBC1D4 (Ser588, Ser318, Ser341, Ser704) (1:1,000, customized by Capra Science, Sweden); AS160/TBC1D4 protein (1:1,000, 07-741; Millipore, Billerica, MA); beta actin (1:1,000, 926-42210; LI-COR Biosciences, Lincoln, NE); GLUT4 (1:1,000, sc-53566; Millipore); GLUT1 (1:1,000, Santa Cruz Biotechnology); peroxisome proliferator-activated receptor γ coactivator α (1:1,000, PGC1α) (ab106814; Abcam); SIRT1 (D739) (1:1,000, 2493; Cell Signaling); and citrate synthase (1:1,000, ab96600; Abcam). Following overnight incubation with primary antibodies, membranes were probed with IRDye secondary antibodies (1:10,000, LI-COR Biosciences) and scanned (Odyssey 9120, LI-COR Biosciences). All of the antibody concentrations were optimized, as presented in other studies (17, 18, 20, 25).

### Statistical Analyses

Unpaired or paired two-tailed Student’s *t* test or two- or three-way ANOVA was used to determine statistical significance. Factors were group (lean vs. severely obese), insulin, and condition (control or resveratrol treatment). Statistical significance was set as *P* ≤ 0.05. When significance was detected for either main effects or interactions between group, insulin, and condition, a two-tailed Student *t* test was performed for distinguishing meaningful differences. All data were expressed as means ± SE.

## RESULTS

### Subject Characteristics

Age-matched lean women and women with severe obesity (BMI ≥ 40 kg/m^2^) were recruited (*n* = 8/group). The women with severe obesity exhibited an elevated BMI, fasting insulin, and HOMA-IR compared with the lean subjects (Table 1).

**Table 1.** Characteristics of the lean and Caucasian women with severe obesity

	Lean	Severely Obese	*P* Value
*n*	8	8	
Sex (male/female)	0/8	0/8	
Ethnicity	8C	8C	
Age, yr	28.8 ± 2.0	32.3 ± 1.8	0.27
BMI, kg/m^2^	21.9 ± 0.7	46.1 ± 3.1	*P* < 0.01
Fasting glucose, mg/dL	85.7 ± 2.26	95.0 ± 7.0	0.21
Fasting insulin, µU/mL	7.0 ± 0.7	15.9 ± 2.0	*P* < 0.01
HOMA-IR	1.5 ± 0.2	3.9 ± 0.8	*P* < 0.01

C, Caucasian. Data are presented as means ± SE, comparisons were made with an unpaired *t* test.

### Resveratrol Enhances Insulin Action in Myotubes

**Figure 1.**
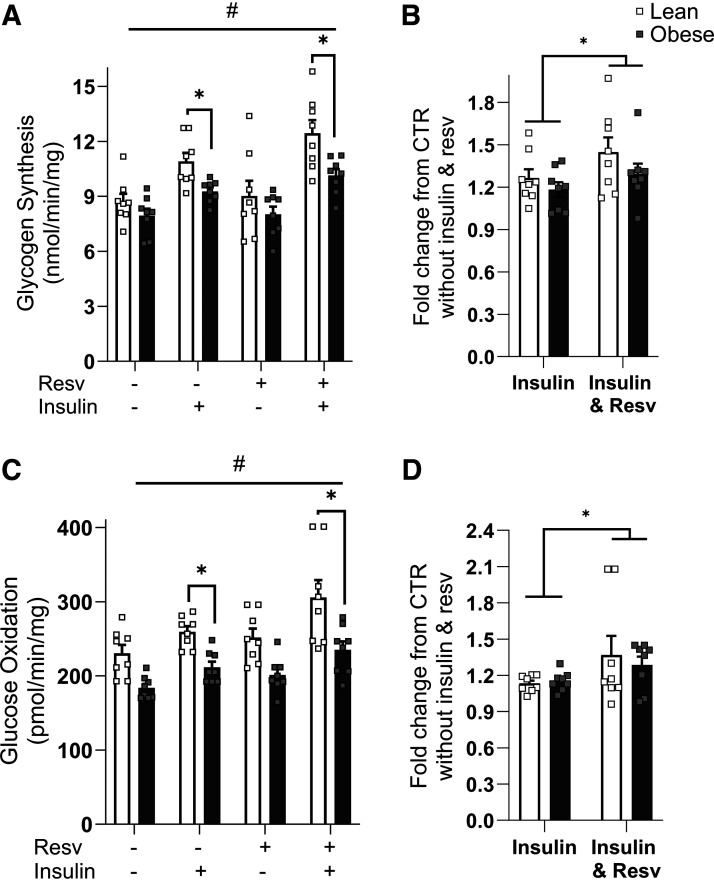
Insulin action in the absence or presence of 100 nM insulin in myotubes from lean and individuals with severe obesity in response to 24 h of resveratrol treatment (Resv). *A*: absolute rates of glycogen synthesis. *B*: fold change in glycogen synthesis over noninsulin, non-Resv condition. *C*: absolute rates of glucose oxidation. *D*: fold change in glucose oxidation over noninsulin, non-Resv condition. Following detecting significance for either group, treatment and/or interaction effect using three-way ANOVA, distinguishing meaningful differences were performed using a two-tailed Student’s *t* test. There were main effects (*P* < 0.05) for insulin and resveratrol for both glycogen synthesis and glucose oxidation. There was a group effect (*P* < 0.05) for insulin-stimulated glycogen synthesis and glucose oxidation. This indicates that resveratrol treatment enhances insulin action in severely obese subjects; however, that did not mitigate the difference between groups. Data are presented as means ± SE. *n* = 8 per group (all females); *P < 0.05 for selected comparison; #*P* < 0.05 for resveratrol. CTR, control.

As presented in Fig. 1*A*, insulin-stimulated glycogen synthesis was reduced in myotubes from women with severe obesity compared with lean donors (10.9 ± 0.5 vs. 9.4 ± 0.2 nmol/min/mg, for lean vs. severely obese, respectively, *P* < 0.05) with and without resveratrol treatment. Resveratrol increased (main treatment effect for resveratrol, *P* < 0.05) both absolute and relative rates (fold-change) of insulin-mediated glycogen synthesis in both cohorts. The relative increase in insulin-stimulated glycogen synthesis with resveratrol over the noninsulin and nonresveratrol condition was enhanced in both groups (*P* < 0.05) compared with insulin alone (Fig. 1*B*). As presented in Fig. 1*C*, insulin-stimulated glucose oxidation was significantly higher in myotubes from lean donors than donors with severe obesity (*P* < 0.05). There was a main treatment effect (*P* < 0.05) for resveratrol for glucose oxidation regardless of participant group. Furthermore, there was a main treatment effect for a relative increase in glucose oxidation with resveratrol treatment (Fig. 1*D*).

### Insulin Signal Transduction

**Figure 2.**
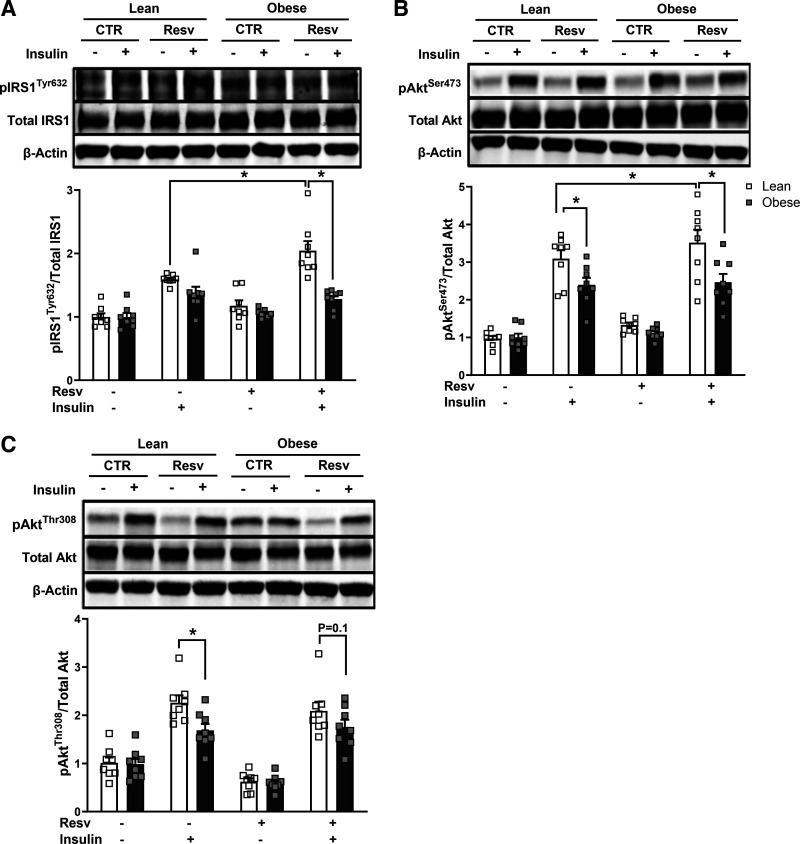
Insulin signal transduction in the absence or presence of 100 nM insulin in myotubes from lean and subjects with severe obesity in response to 24 h of resveratrol treatment (Resv). *A*: IRS1 (Tyr632) phosphorylation. *B*: Akt (Ser473) phosphorylation. *C*: Akt (Thr308) phosphorylation. Following detecting significance for either group, treatment and/or interaction effect using three-way ANOVA, distinguishing meaningful differences were performed using a two-tailed Student’s *t* test. There were main effects (*P* < 0.05) for insulin and resveratrol for all the proteins and for subject group for pIRS1 and pAkt (Ser473). There were interaction effects (*P* < 0.05) for subject group with resveratrol for pIRS1 and pAkt (Ser473) and for subject group with insulin for pIRS1. Data are presented as means ± SE. *n* = 8 per group (all females); *P < 0.05 for selected comparison. CTR, control.

As presented in Fig. 2*A*, insulin-stimulated IRS1 (Tyr632) phosphorylation increased with resveratrol treatment in lean subjects but not in subjects with severe obesity (1.9 ± 0.2 vs. 1.3 ± 0.1 fold increase over the noninsulin, nonresveratrol-treated condition, for lean vs. severely obese, respectively, *P* < 0.05). As presented in Fig. 2*B*, insulin-stimulated Akt (Ser473) phosphorylation was higher in lean subjects than subjects with obesity, and insulin-stimulated Akt pSer473 phosphorylation further increased with resveratrol in lean subjects but not in subjects with severe obesity (*P* < 0.05). In addition, insulin-stimulated Akt (Thr308) phosphorylation was significantly elevated in lean subjects (2.3 ± 0.2 vs. 1.7 ± 0.2 fold increase over noninsulin, nonresveratrol-treated condition, for lean vs. severely obese, respectively, *P* < 0.05). Resveratrol elicited no changes in insulin-stimulated pThr308 in both groups, whereas resveratrol tended to reduce the difference between lean women versus women with severe obesity (*P* = 0.10).

**Figure 3.**
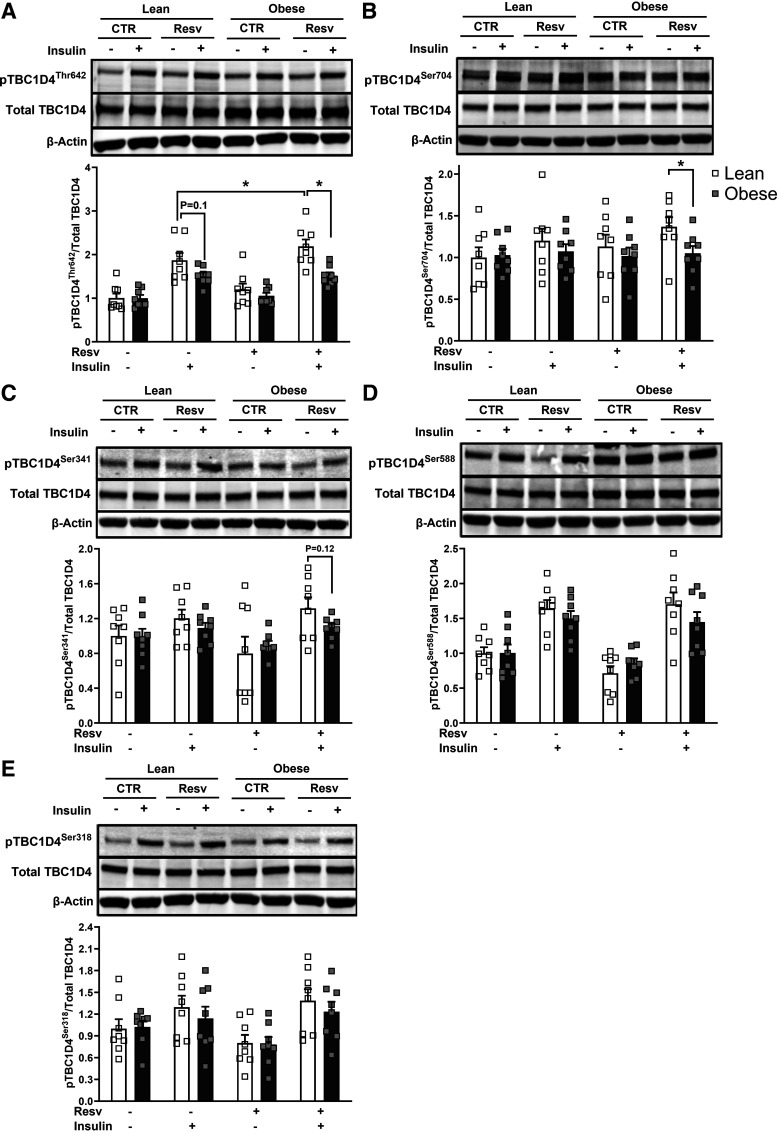
Signatures on TBC1D4 phosphorylation in the absence or presence of 100 nM insulin in myotubes from lean and subjects with severe obesity in response to 24 h of resveratrol treatment (Resv). *A*: Thr642 phosphorylation. *B*: Ser704 phosphorylation. *C*: Ser341 phosphorylation. *D*: Ser588 phosphorylation. *E*: Ser318 phosphorylation. Following detecting significance for either group, treatment, and/or interaction effect using three-way ANOVA, distinguishing meaningful differences were performed using a two-tailed Student’s *t* test. There were main effects (*P* < 0.05) for insulin for all measured sites of pTBC1D4s and for resveratrol for pThr642. There was a group effect with resveratrol for pThr642 and pSer704. Data are presented as means ± SE. *n* = 8/group (all females); *P < 0.05 for selected comparison. CTR, control.

The phosphorylation signatures of TBC1D4 showed different responses to resveratrol treatment (Fig. 3). Insulin-stimulated TBC1D4 (Thr642) phosphorylation exhibited a trend to be repressed with obesity (*P* = 0.10); this difference became more evident after resveratrol treatment in pThr642 and pSer704 (*P* < 0.05) (Fig. 3, *A*–*C*). As presented in Fig. 3*A*, insulin-stimulated pThr642 with resveratrol significantly increased only in lean subjects (1.7 ± 0.3 vs. 2.2 ± 0.3 fold increase over the noninsulin, nonresveratrol-treated condition for insulin vs. insulin with resveratrol, respectively, *P* < 0.05). Resveratrol treatment resulted in no changes in insulin-stimulated pSer588 and pSer318 in both groups. There were no differences in either GLUT1 or GLUT4 protein content between the subject groups with or without resveratrol treatment (Supplemental Fig. S1, *A* and *B*).

### Resveratrol Improved Expression of pAMPK, but not SIRT1, PGC1α, and Citrate Synthase in Severe Obesity

**Figure 4.**
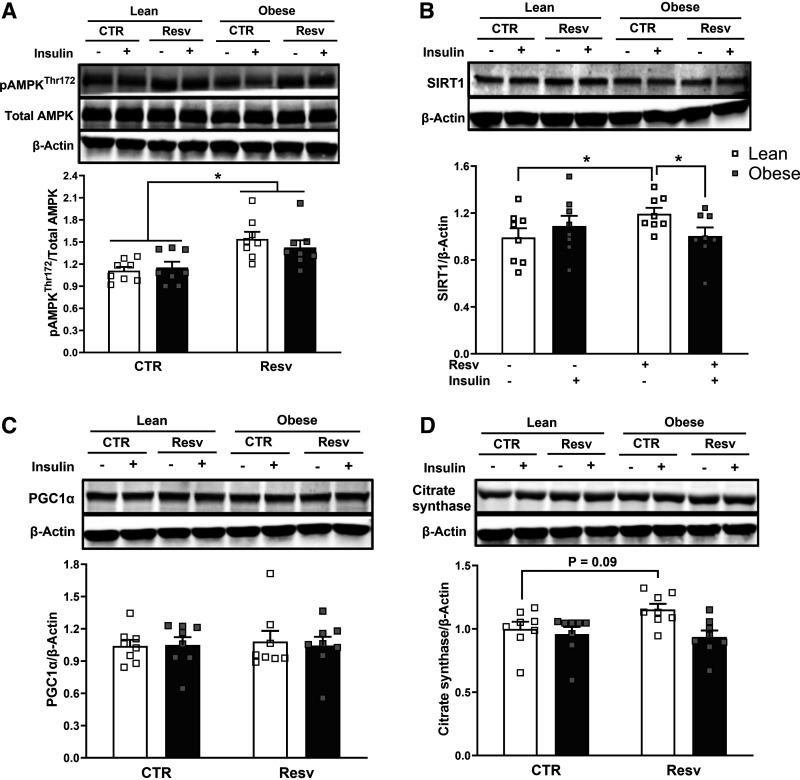
AMPK(Thr172) phosphorylation, SIRT1, PGC1α, citrate synthase protein content in myotubes from lean and severely obese subjects in response to 24 h of resveratrol treatment (Resv). *A*: AMPK (Thr172) phosphorylation. *B*–*D*: SIRT1, PGC1α, and citrate synthase protein content. Following detecting significance for either group, treatment, and/or interaction effect using three-way ANOVA, distinguishing meaningful differences were performed using a two-tailed Student’s *t* test. There were main effects (*P* < 0.05) for resveratrol for pAMPK(Thr172) for both groups. There was a significant interaction effect (*P* < 0.05) for subject group with resveratrol for SIRT1. Data are presented as means ± SE. *n* = 8 per group (all females); *P < 0.05 for selected comparison. CTR, control.

Resveratrol significantly increased AMPK phosphorylation in myotubes from both groups (main treatment effect for resveratrol) (1.3 ± 0.1 and 1.2 ± 0.1 fold increase over the nonresveratrol condition, respectively, *P* < 0.05) (Fig. 4*A*). In contrast, as presented in Fig. 4*B*, SIRT1 protein content increased with resveratrol in only myotubes from lean subjects (1.2 ± 0.04 fold increase over the nonresveratrol condition, *P* < 0.05) and changed enough to be significantly higher in lean participants than participants with severe obesity (*P* < 0.05). PGC1α protein content did not differ between groups nor change with resveratrol (Fig. 4*C*). Similarly, citrate synthase content did not change following resveratrol incubation in either group (Fig. 4*D*), although there was a trend to increase in the lean subjects (*P* = 0.09). Data supplements can be accessed here: https://doi.org/10.6084/m9.figshare.25078115.v1.

## DISCUSSION

Skeletal muscle is the tissue responsible for the majority of insulin-mediated postprandial glucose disposal (26). The main finding of this study was that resveratrol treatment improved insulin-mediated glucose metabolism in myotubes from lean individuals and/or individuals with severe obesity. As anticipated, myotubes from donors with severe obesity were initially insulin resistant (Fig. 1); however, despite this impairment, there was a main treatment effect for resveratrol indicating enhanced insulin action.

The ability of resveratrol treatment to improve the insulin resistance is supported by animal studies reporting improvements in insulin sensitivity (5, 9) and insulin signal transduction (10, 27). In mice, resveratrol was able to attenuate the insulin resistance induced by a high-fat diet (10, 28). In humans, Timmers et al. (7) reported that 30 days of resveratrol supplementation (150 mg/day) in women with obesity significantly decreased HOMA-IR, an index of insulin resistance.

In the present study, resveratrol improved insulin-mediated signal transduction [insulin-stimulated phosphorylation of IRS1 (Tyr632), Akt (Ser473), and TBC1D4 (Thr642)] in the myotubes from lean individuals, but not in the myotubes from individuals with severe obesity (Figs. 2 and 3). This finding in the lean subjects is similar to that reported in C2C12 cells, where insulin-stimulated glucose uptake and Akt phosphorylation increased with resveratrol treatment (10). In addition, Sun et al. (10) reported an increase in Akt phosphorylation with resveratrol in C2C12 cells initially treated with lipid to induce insulin resistance; however, in our insulin-resistant individuals with severe obesity, we did not observe improvements in the insulin-signaling cascade, which suggests that the resveratrol-mediated mechanisms involved in improving insulin action may differ between lipid exposure and tissue that is intrinsically insulin resistant.

In the present study, resveratrol-induced improvements in insulin action in myotubes from donors with severe obesity occurred in the absence of changes in insulin signaling. Our findings indicate that resveratrol potentiates TBC1D4 (Thr642) phosphorylation in myotubes from only lean subjects, while none of the phosphorylation sites of TBC1D4 exhibited improvement in response to resveratrol treatment in myotubes from severely obese women. The findings in myotubes from lean donors is in agreement with an exercise study in healthy lean humans where knee extension for 1 h enhanced pTBC1D4 (Thr642, Ser704, and Ser341) (29, 30); however, 1 h of cycling at 65% V̇o_2max_ increased pTBC1D4 (Ser704, Ser341 but not Thr642) (31). These data indicate that resveratrol can produce exercise-mimicking effects in the phosphorylation signatures of TBC1D4, but this response may be exercise-mode dependent and only evident in lean individuals. The data presented here as well as those from others (9, 10, 27) suggest that in individuals with obesity, resveratrol treatment can be sufficient in ameliorating insulin resistance independent of improvement in insulin signal transduction. However, further studies both in vivo and in vitro are needed to test this hypothesis.

Previous findings indicate that resveratrol induces AMPK phosphorylation and increases SIRT1 and PGC1α content, leading to an improvement in metabolic function (5, 32). However, the effects of resveratrol are complex and involve the activation of an array of metabolism-relevant signaling pathways (33, 34). Um et al. (11) concluded, using AMPK knockout mice, that AMPK was the central target for the metabolic effects of resveratrol. Similarly, in the present study resveratrol activated AMPK phosphorylation in human myotubes regardless of obesity status (Fig. 4*A*). In addition, a well-established effect of AMPK activation is to enhance glucose uptake (32), which may explain the improvement in insulin action we observed in both lean individuals and individuals with severe obesity (Fig. 1); however, this necessitates further investigation. An intriguing finding was that resveratrol treatment in women with severe obesity increased insulin action and AMPK phosphorylation without changing other components in the AMPK signaling network, such as SIRT1 and PGC1α. This is in agreement with a previous study reporting that resveratrol supplementation for 12 wk in insulin-resistant mice (high-fat diet) improved insulin action in conjunction with enhanced AMPK phosphorylation and PGC1α expression but without an increase in SIRT1 (11). However, we acknowledge that a limitation of the current experimental approach was not examining other potential targets that could have supported or refuted these observations. The current and previous data thus suggest that the activation of AMPK with resveratrol potentially plays a role in enhancing glucose metabolism. AMPK activation has been reported to promote the translocation of the insulin-sensitive GLUT4 pool (33), which would enhance insulin action independently of changes in insulin signal transduction and should be further explored in individuals with obesity.

In conclusion, resveratrol appears to provide beneficial effects in insulin-stimulated glucose metabolism in myotubes from lean individuals and individuals with severe obesity. Insulin-stimulated glycogen synthesis, glucose oxidation, and insulin signal transduction were blunted in the severely obese individuals. However, resveratrol was sufficient to improve insulin-stimulated glucose metabolism irrespective of donors phenotype (lean or severely obese). The improvement in insulin action occurred in parallel with the higher activation of AMPK in the skeletal muscle of individuals with severe obesity.

## DATA AVAILABILITY

Data will be made available upon reasonable request.

## SUPPLEMENTAL DATA

10.6084/m9.figshare.25078115.v1 Supplemental Data S1 and Supplemental Fig. S1: https://doi.org/10.6084/m9.figshare.25078115.v1.

## GRANTS

This work was supported by the Gachon University research fund of 2023 (GCU-202305210001). J.T.T. was supported by the Novo Nordisk Foundation Center for Basic Metabolic Research (CBMR). CBMR is an independent Research Center at the University of Copenhagen, which is funded by an unrestricted donation from the Novo Nordisk Foundation (NNF18CC0034900).

## DISCLOSURES

No conflicts of interest, financial or otherwise, are declared by the authors.

## AUTHOR CONTRIBUTIONS

S.P. and J.A.H. conceived and designed research; S.P. performed experiments; S.P. analyzed data; S.P. interpreted results of experiments; S.P. prepared figures; S.P. drafted manuscript; S.P., F.J., P.M.K., A.B.C., D.Z., J.T.T., and J.A.H. edited and revised manuscript; F.J. and J.A.H. approved final version of manuscript.
